# Anaplastic histology and distinct molecular features in a small series of spinal cord ependymomas

**DOI:** 10.1007/s00401-024-02740-y

**Published:** 2024-05-12

**Authors:** Ulrich Schüller, Antonia Gocke, Shweta Godbole, Claire Delbridge, Christian Thomas, Julia E. Neumann

**Affiliations:** 1 Department of Pediatric Hematology and Oncology, University Medical Center Hamburg-Eppendorf, Hamburg, Germany; 2 Research Institute Children’s Cancer Center Hamburg, Hamburg, Germany; 3 Institute of Neuropathology, University Medical Center Hamburg-Eppendorf, Hamburg, Germany; 4 Center for Molecular Neurobiology Hamburg (ZMNH), University Medical Center Hamburg-Eppendorf, Hamburg, Germany; 5 Section of Mass Spectrometric Proteomics, University Medical Center Hamburg-Eppendorf, Hamburg, Germany; 6 Institute of Pathology, Department of Neuropathology, TUM School of Medicine and Health, Technical University Munich, Munich, Germany; 7 Institute of Neuropathology, University Hospital Münster, Münster, Germany

Ependymomas belong to the most frequent intramedullary tumors of the spinal cord and occur at all ages. These tumors encompass a broad heterogeneity with multiple types and subtypes, each of them harboring distinct histological, molecular, and clinical features [1, 4, 6]. Apart from the well-known subependymomas (CNS WHO grade 1), ependymomas, and myxopapillary ependymomas (both CNS WHO grade 2), spinal ependymomas with *MYCN* amplification have more recently been identified as the most aggressive type of spinal ependymoma [3]. Of note, the latter includes most of the spinal tumors that had been diagnosed as ‘anaplastic ependymoma (WHO grade III)’ in the pre-molecular era. While amplifications of *MYCN* therefore need to be investigated specifically, global DNA methylation profiling has emerged as an extremely valuable tool to classify tumors of the central nervous system in general and ependymomas specifically [2, 7].

Here, we describe a series of seven unusual ependymomas that mostly occurred in the spinal cord of adult patients (Fig. 1a). Tumor tissue was available from cases 1–4, all of which displayed anaplastic features by histology, including nuclear pleomorphism, microvascular proliferation, and mitotic figures (Fig. 1b, Suppl. Figure 1). Apart from expression of GFAP, these tumors also displayed OLIG2 and MYCN expression, together with a high proliferative activity as shown by Ki67 staining (Fig. 1c–f, Suppl. Figure 1). As revealed by global DNA methylation profiling, all seven cases showed similar epigenetic profiles that were distinct from various other central nervous system tumors including spinal ependymomas and subependymomas (Suppl. Figure 2, Fig. 1g, Suppl. Figure 3). DKFZ-based brain tumor classification (https://www.molecularneuropathology.org, [1]) suggested a similarity of some of the cases to subependymomas (depending on its version, Fig. 1a), but epigenetics and histology were clearly distinct from spinal subependymomas (Fig. 1b–g, Suppl. Figure 1–4). Copy number profiles for all 7 here described spinal ependymomas were inferred from global DNA methylation data. Chromosomal gains and losses were different from other types of ependymal tumors in the spinal cord with losses on chromosome 6 as the most prominent aberration (Suppl. Figure 5). Next-generation sequencing revealed *TERT* and *PIK3R1* alterations in case 1, whereas whole-exome sequencing did not disclose relevant alterations of tumor-related genes in cases 2–4 (Fig. 1a). Follow-up data were available for three patients, ranging from 2–21 years without events.

**Fig. 1:**
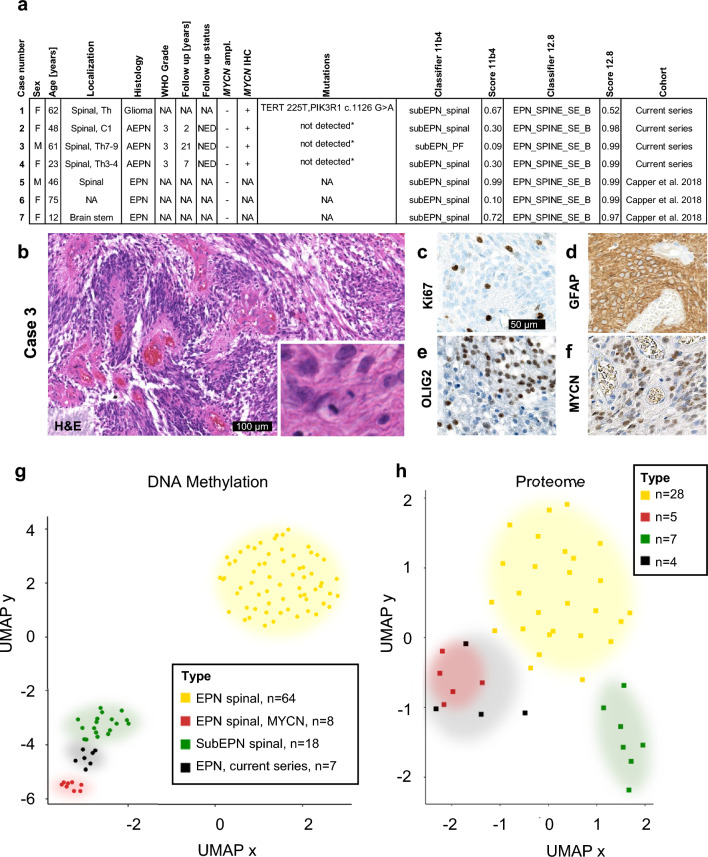
Clinical features, histology, and molecular profiles of spinal ependymomas with distinct neuropathological features. Details for each of the seven cases are noted in (**a**). H&E histology is shown in (**b**) for case 3 with perivascular pseudorosettes, a high cell density, pleomorphic tumor cell nuclei, and mitotic figures (inset). Immunohistochemistry reveals expression of GFAP (**c**), OLIG2 (**d**), MYCN (**e**), and Ki67 (**f**) in a large fraction of tumor cells. *T*-distributed neighbor embedding (*t*-SNE) of global DNA methylation data demonstrates the distinct molecular profile of the here described seven cases (**g**), which is confirmed by proteomic analyses (**h**). *AEPN* anaplastic ependymoma, *EPN* ependymoma, *NA* not available, *NED* no evidence of disease. *no relevant variant detected upon whole-exome sequencing

The distinct identity of our series was finally confirmed by mass spectrometric proteomic analyses of the four cases, for which tumor material was available (Fig. 1h, Suppl. Table 1), quantifying 5,878 proteins. Unsupervised analysis of spinal ependymal tumors revealed a similarity of the described samples with proteome patterns of spinal *MYCN*-amplified ependymomas but clear distances from spinal ependymomas and subependymomas (Fig. 1h).

Although our cases had anaplastic features and showed expression of MYCN protein by immunohistochemistry, *MYCN* amplifications were neither detectable by FISH (n = 4) nor by whole genome copy number profiles, although case 7 showed a slightly elevated, diagnostically unclear signal at the *MYCN* locus (Suppl. Figure 5). We therefore conclude that the here described cases fall into a previously undescribed group of distinct spinal ependymomas. As visible from the histology of all four cases with available tissue within this series, the designation as subependymomas (as defined by the WHO [5]) appears inappropriate, and we propose the provisional designation as MYCN-like spinal ependymoma (SP-EPN-MYCN-like). Larger series of such cases, together with more in-depth investigations, are warranted to uncover e. g. genetic drivers of the tumors and clinical outcomes of respective patients.

### Supplementary Information

Below is the link to the electronic supplementary material.

Supplementary file1 (PDF 1376 kb)

Supplementary file2 (XLSX 381 kb)

## Data Availability

DNA methylation data are available via GEO accession number GSE264714.
