# Supplementary material for: Anaplastic histology and distinct molecular features in a small series of spinal cord ependymomas
Source: Acta Neuropathol. 2024 May 12;147(1):83. doi: 10.1007/s00401-024-02740-y (PMC11089008; doi:10.1007/s00401-024-02740-y)
Supplement: Supplementary file 1 — Supplementary file1 (PDF 1376 kb) [file 401_2024_2740_MOESM1_ESM.pdf]

Schüller et al., Suppl. Figure 1

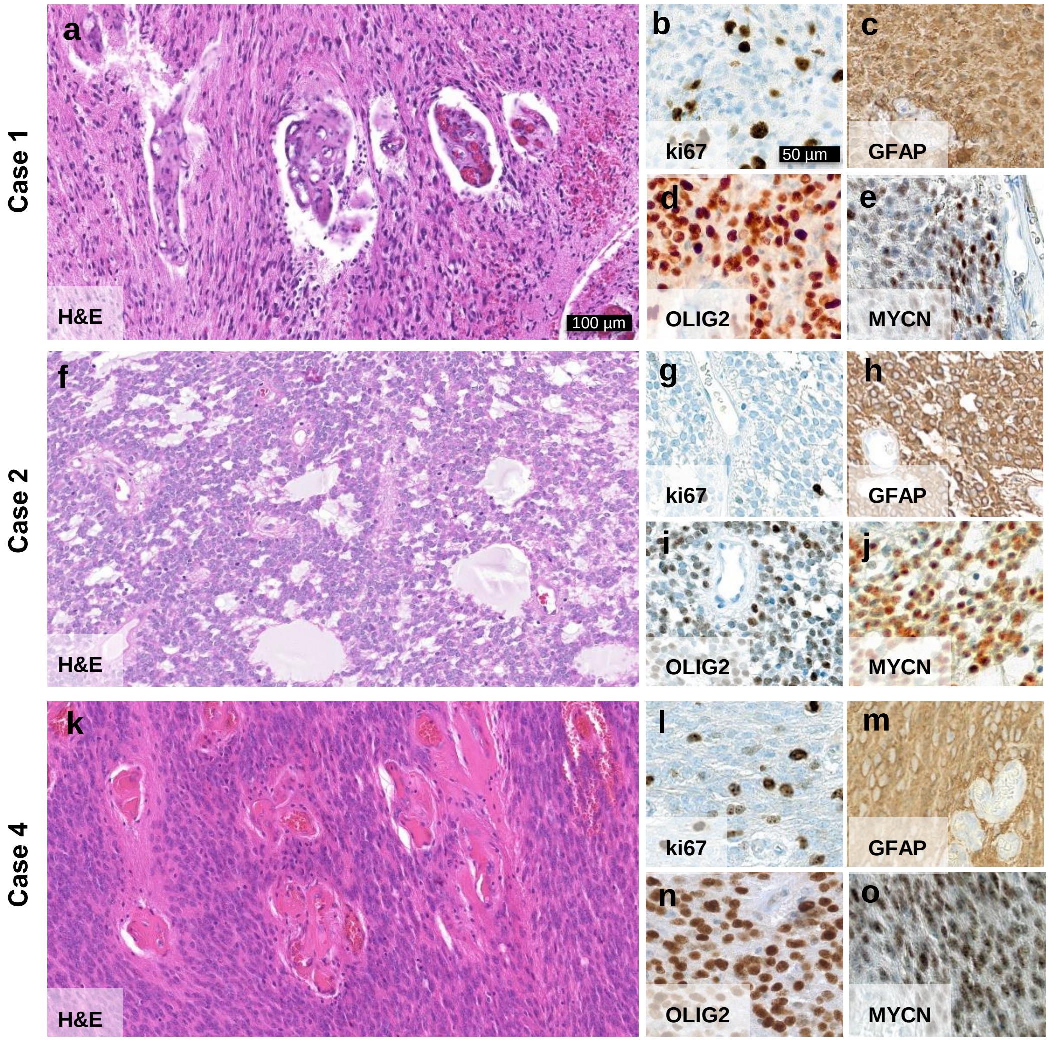

Suppl. Figure 1: Histological and immunohistochemical features of cases 1, 2, and 4.

Schüller et al., Suppl. Figure 2

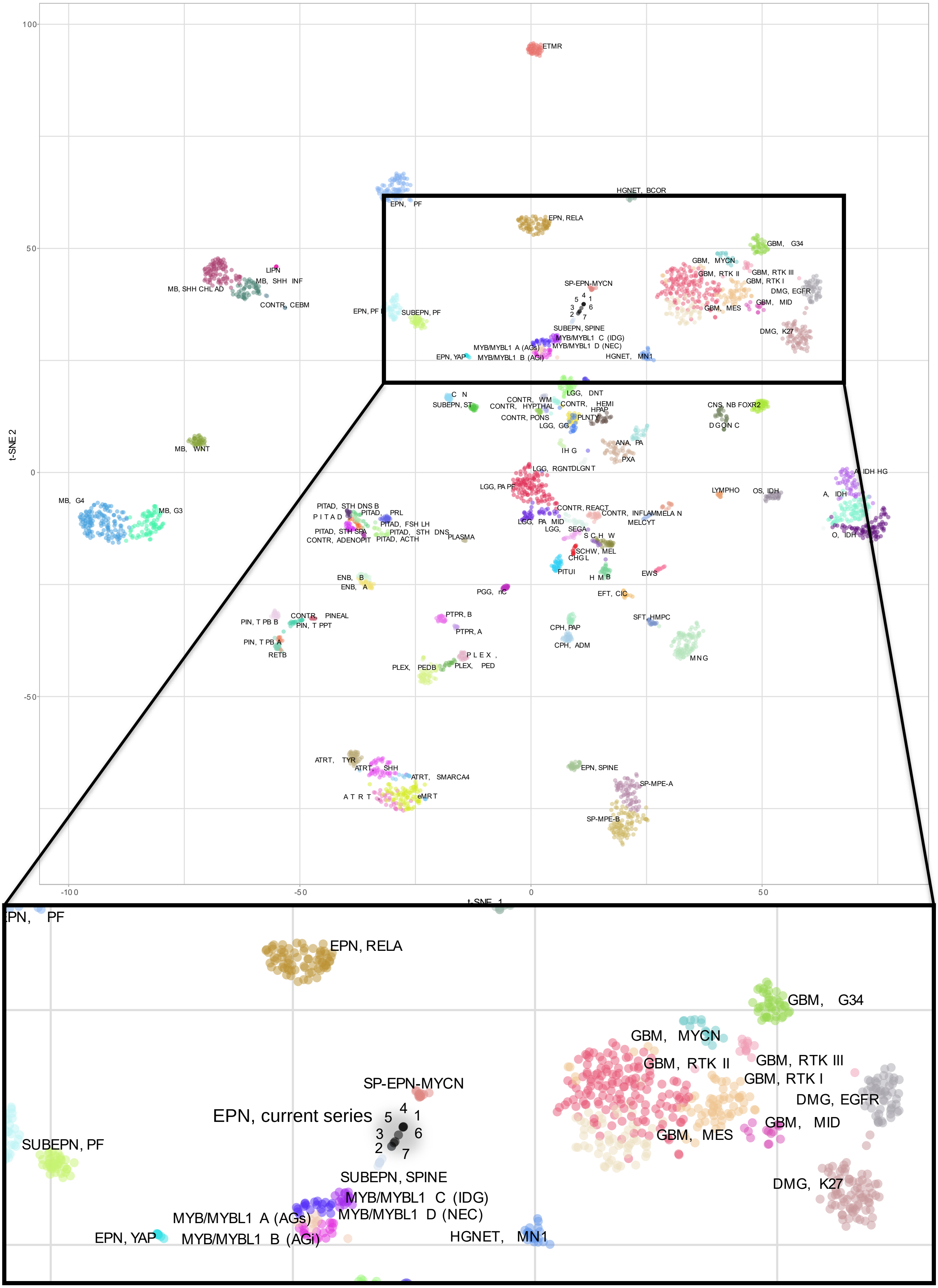

Suppl. Figure 2: Global DNA methylation profiling of brain tumor reference cases (Capper et al. 2018) and all 7 cases of the here described spinal ependymomas shown as *t*-SNE.

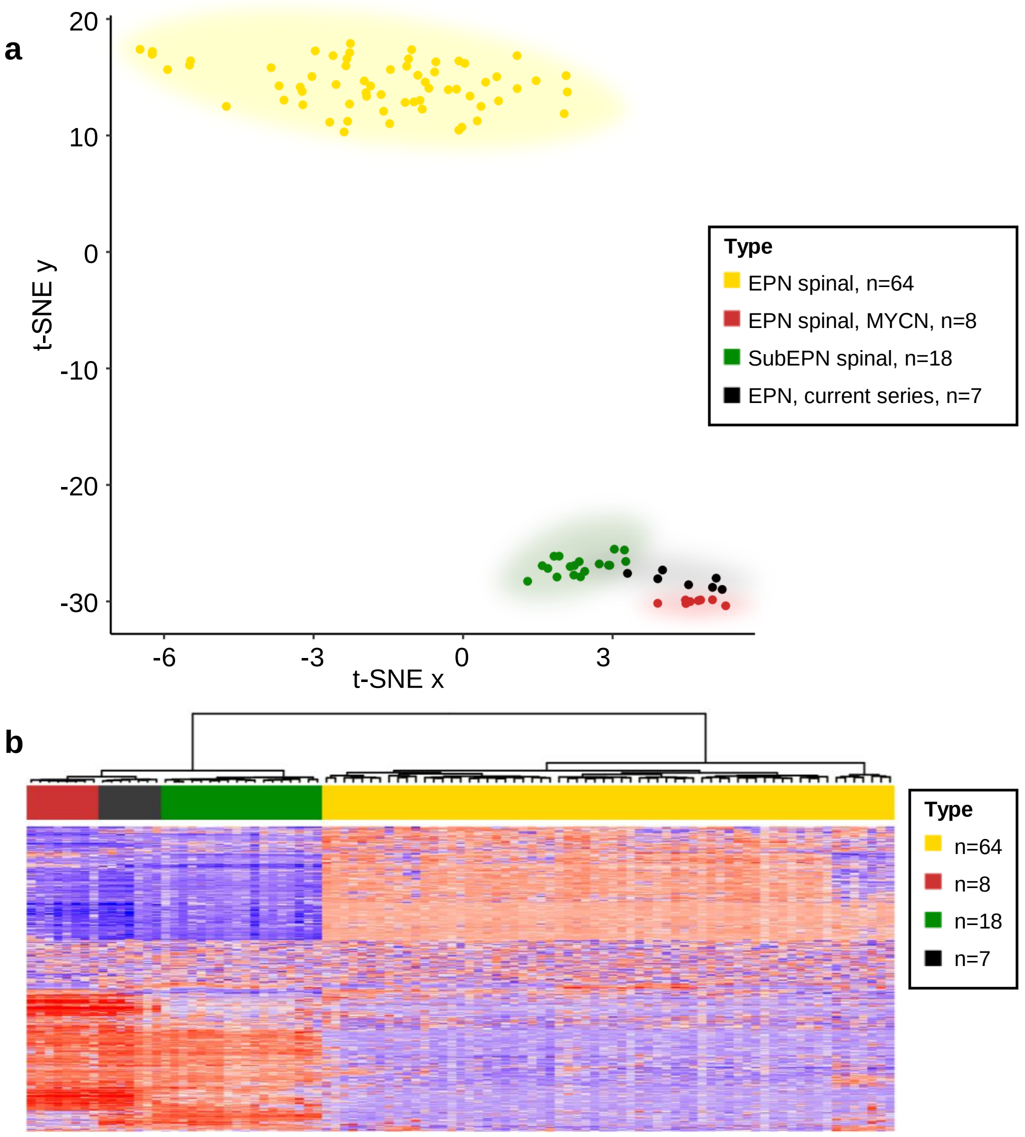

Suppl. Figure 3: t-SNE analysis (**a**) and unsupervised hierarchical clustering (**b**) based on the 2,000 most variable CpG sites confirm the epigenetic distinctivness of the here described 7 cases.

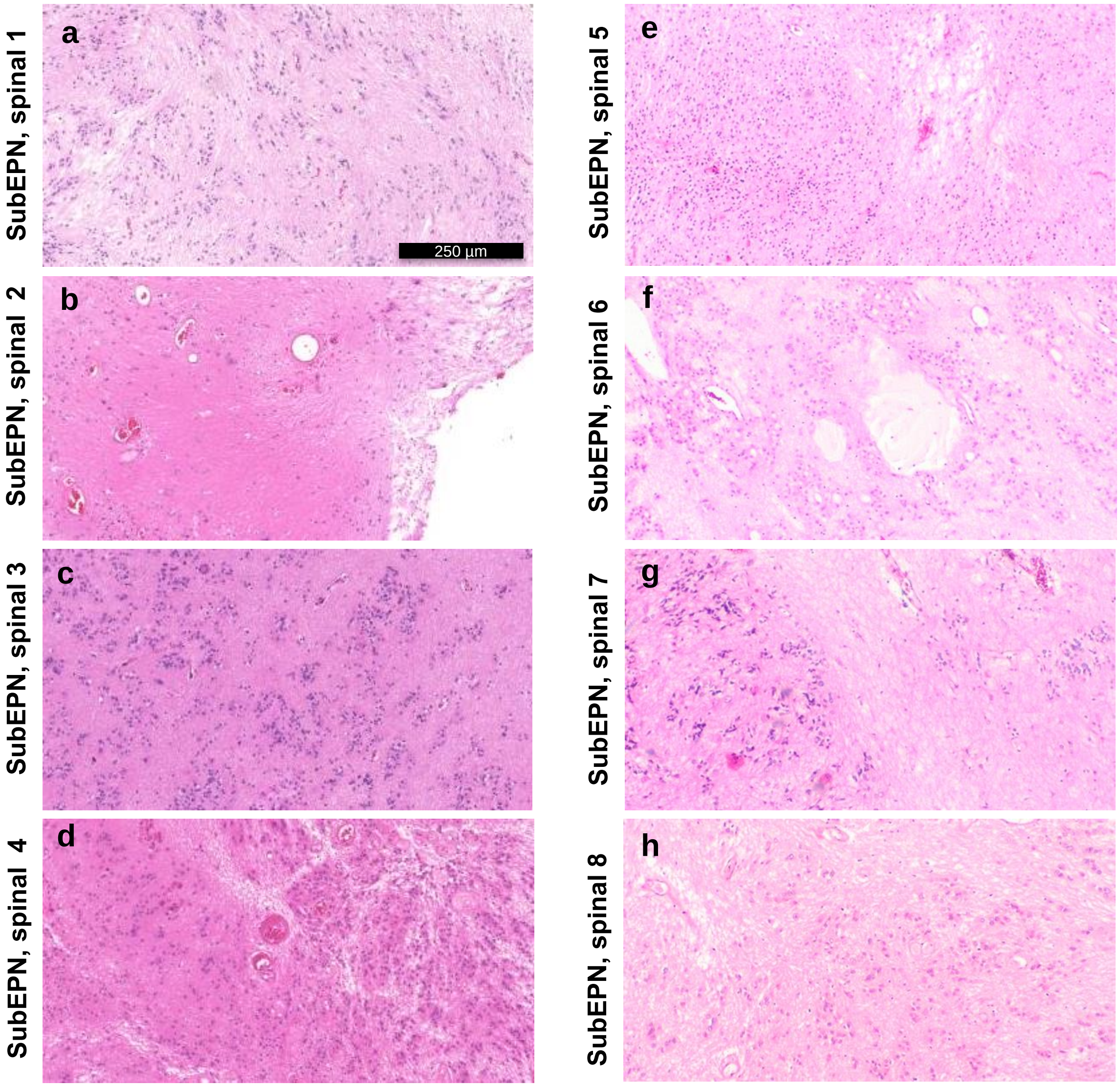

Suppl. Figure 4: Histological features of 8 cases of conventional spinal subependymoma (SubEPN), which fall apart from the here described series of spinal ependymoma by histology and global DNA methylation.

Schüller et al., Suppl. Figure 5

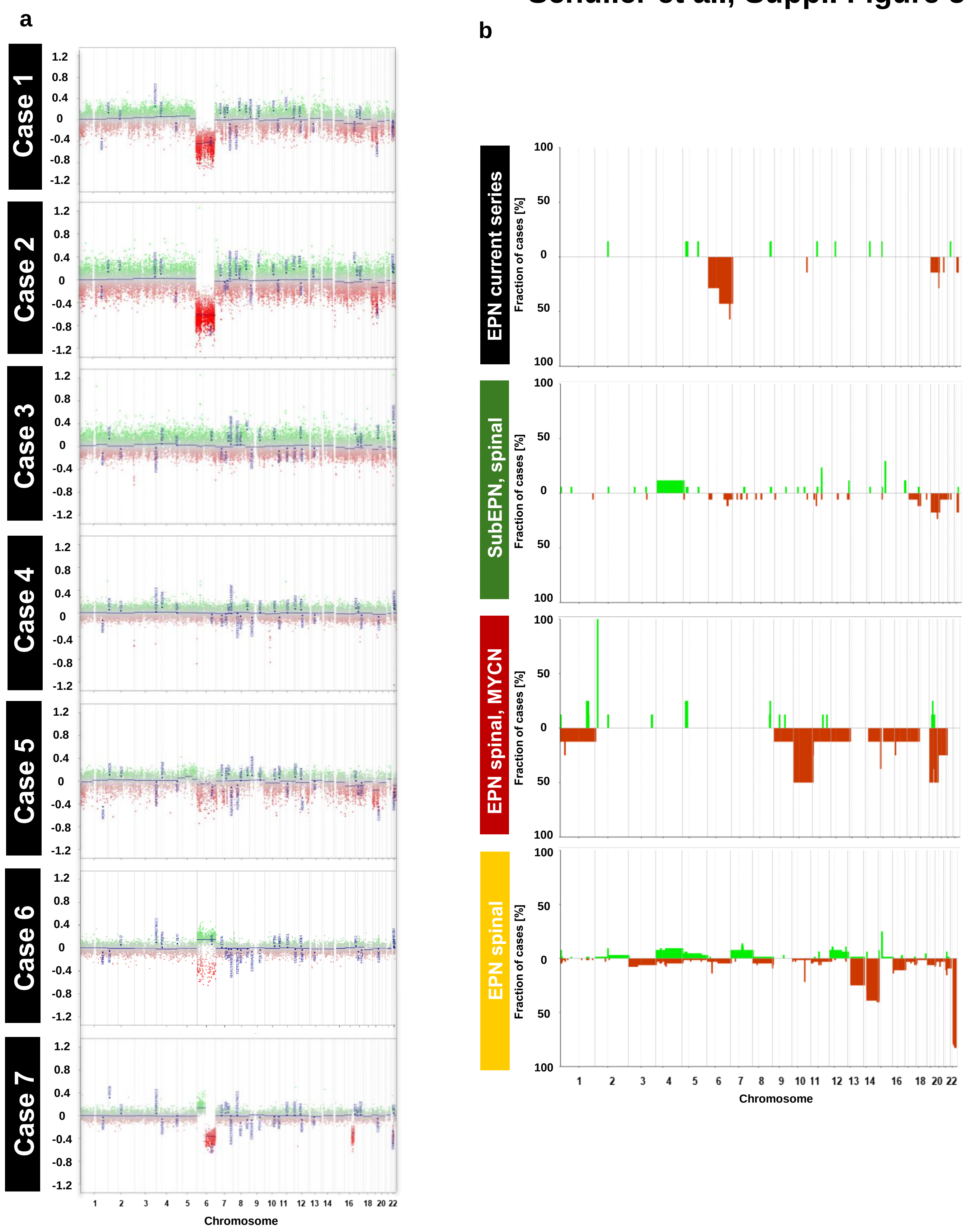

Suppl. Figure 5: Copy number profiles of all 7 cases of the here described series of spinal ependymoma (**a**). Cumulative copy number plots confirm loss of chromosome 6 as most common abberation and reveal differences to other types of ependymomal tumors in the spinal cord (**b**).
